# Correlates of missed or late versus timely diagnosis of dementia in healthcare settings

**DOI:** 10.1002/alz.091731

**Published:** 2025-01-09

**Authors:** Yi Chen, Melinda Power, Francine Grodstein, Ana W. Capuano, Brittney S Lange‐Maia, Ali Moghtaderi, Emma K Stapp, Joya Bhattacharyya, Raj C. Shah, Lisa L. Barnes, David A. Bennett, Bryan D James

**Affiliations:** ^1^ Rush Alzheimer’s Disease Center, Chicago, IL USA; ^2^ George Washington University, Washington DC, DC USA; ^3^ Rush University Medical Center, Chicago, IL USA; ^4^ The George Washington University, Washington, DC USA; ^5^ Department of Psychiatry and Behavioral Sciences, Rush University Medical Center, Chicago, IL USA; ^6^ Department of Neurological Sciences, Rush University Medical Center, Chicago, IL USA; ^7^ Department of Neurological Sciences, Rush Medical College, Chicago, IL USA; ^8^ Department of Internal Medicine Rush University Medical Center, Chicago, IL USA

## Abstract

**Background:**

Previous research suggests dementia is undetected in healthcare settings for more than half of those with dementia. Most research on underdiagnosis of dementia has focused on the presence or absence of a clinical diagnosis in claims or the medical record without addressing differences in the timeliness of a diagnosis. Little is known about factors that may be related to the timeliness of clinical dementia diagnosis.

**Method:**

In five longitudinal cohorts at Rush Alzheimer’s Disease Center, we identified participants with incident dementia based on annual research assessments from 1994 to 2019 and examined the timing of dementia diagnoses in Medicare claims. Receiving a claims diagnosis within three years prior to or one year following incident dementia was considered a “timely diagnosis”; the remaining were categorized as “underdiagnosis”, including late or missed diagnosis. We assessed correlates of timely versus under‐ diagnosis, including sociodemographic characteristics, health, and psychosocial factors, using bivariate analyses and adjusted logistic regressions.

**Result:**

Of the 710 participants with incident dementia, 54% (n = 385) received a timely diagnosis. In logistic regressions adjusting for demographic characteristics, we found Black older adults had twice the odds of underdiagnosis versus timely diagnosis (OR = 2.15, 95% CI: 1.21‐3.82). Higher mid‐life income (on 10‐level scale) was related to 11% lower odds of underdiagnosis (OR = 0.89, 95% CI: 0.81‐0.97). Better cognitive function (OR = 1.48, 95% CI: 1.10‐1.98 for one standard‐deviation higher cognitive function), and fewer comorbidities (OR = 0.94, 95% CI: 0.89‐0.98 for one‐unit increase in Elixhauser Comorbidity Index) at the time of dementia onset were associated with diagnostic delay. The number of encounters with the healthcare system, disability, frailty, social network, psychosocial factors, and APOE‐4 genotype were not associated with diagnostic timing.

**Conclusion:**

When considering the timeliness of dementia diagnosis, two socio‐demographic characteristics (Black race and lower income) and two health characteristics (better cognitive function and fewer comorbidities) were related to receiving a late/missed diagnosis. Observed correlates of timeliness may have implications for improving dementia diagnosis and care.

